# Dynamic of centromere associated RNAs and the centromere loading of DNA repair proteins in growing oocytes

**DOI:** 10.3389/fgene.2023.1131698

**Published:** 2023-03-24

**Authors:** Lin-Li Yang, Yan-Chu Li, Tian-Jin Xia, Sen Li, Xie Feng, Chao Li, Feng-Yun Xie, Xiang-Hong Ou, Jun-Yu Ma

**Affiliations:** ^1^ The Second School of Clinical Medicine, Southern Medical University, Guangzhou, China; ^2^ Fertilization Preservation Lab, Guangdong-Hong Kong Metabolism and Reproduction Joint Laboratory, Guangdong Second Provincial General Hospital, Guangzhou, China; ^3^ College of Life Sciences, Qingdao Agricultural University, Qingdao, China; ^4^ State Key Laboratory of Stem Cell and Reproductive Biology, Institute of Zoology, Chinese Academy of Sciences, Beijing, China

**Keywords:** MRE11, PRKDC, MLH1, oocyte, centromere associated RNAs

## Abstract

Mammalian centromeres are generally composed of dispersed repeats and the satellites such as α-satellites in human and major/minor satellites in mouse. Transcription of centromeres by RNA polymerase II is evolutionary conserved and critical for kinetochore assembly. In addition, it has been found that the transcribed satellite RNAs can bind DNA repair proteins such as MRE11 and PRKDC, and excessively expressed satellite RNAs could induce genome instability and facilitate tumorigenesis. During the maturation of female oocyte, centromeres are critical for accurate segregation of homologous chromosomes and sister chromatids. However, the dynamics of oocyte centromere transcription and whether it associated with DNA repair proteins are unknown. In this study, we found the transcription of centromeres is active in growing oocytes but it is silenced when oocytes are fully grown. DNA repair proteins like Mlh1, Mre11 and Prkdc are found associated with the minor satellites and this association can be interfered by RNA polymerase II inhibitor α-amanitin. When the growing oocyte is *in vitro* matured, Mlh1/Mre11/Prkdc foci would release from centromeres to the ooplasm. If the oocytes are treated with Mre11 inhibitor Mirin, the meiosis resumption of growing oocytes with Mre11 foci can be suppressed. These data revealed the dynamic of centromeric transcription in oocytes and its potential association with DNA repair proteins, which provide clues about how oocytes maintain centromere stability and assemble kinetochores.

## Introduction

Among species from yeast to human, centromeres play critical roles in both mitotic and meiotic chromosome segregation, however, the DNA sequences constituting centromeres are extremely diverse (Muller et al., 2019). For animals and plants, centromeres and peri-centromeres are generally composed by the species specific tandem head-to-tail arrays of repeats, termed satellites, and the dispersed elements (Plohl et al., 2014). In human, centromeric satellite DNA are composed of the alpha-satellites (hSATa), whereas mouse satellite DNA are composed of major and minor satellites (majSAT and minSAT) (Muller et al., 2019). These core centromeres composed of satellites are organized in nucleosomes by centromere specific histone CENP-A and H3. CENP-A can bind with inner kinetochore proteins such as CENP-C throughout the cell cycle. When cell is going to enter mitosis, inner kinetochore proteins will further recruit proteins to construct the outer kinetochore by which centromeres can be linked with micro-tubulins (Navarro and Cheeseman, 2021). Unlike the non-centromeric satellites or the silenced centromeres on chromosome arms, satellites in active centromeres are transcription active (Talbert and Henikoff, 2018). The transcription of centromeric satellites is mediated by RNA polymerase II (RNAPII) and critical for the assembly of kinetochores for correct chromosome segregation (Talbert and Henikoff, 2018). It has been reported that both transcription inhibition of centromere satellites and knockdown of centromeric RNAs will reduce the incorporation of CENP-A in centromeres (Chueh et al., 2009; Bergmann et al., 2011). In addition, over-expression of human hSATa or mouse majSAT in cells could induce genome instability by impairing DNA replication (Zhu et al., 2018). These results suggested that uncontrollable centromeric transcription might not only harm kinetochore assembly but also induce genome instability, although the detailed biological functions of centromeric transcription and the centromeric transcripts remain poorly understood.

Excessive expression of centromeric satellites are common in cells of human and mouse cancers such as breast cancers and pancreatic ductal adenocarcinomas (Ting et al., 2011; Zhu et al., 2018). Overexpressed satellite RNAs can bind with a plenty of proteins associated with DNA replication and repair (Zhu et al., 2011; Zhu et al., 2018). In these satellite RNA-binding proteins, MRE11 complexed with RAD50 and NBS1 initiates the homologous recombination repair of DNA double-strand breaks (DSBs) by end resection (Zhao et al., 2020), and PRKDC is critical for the non-homologous end joining repair of DSBs (Chen et al., 2021). In addition to centromeric RNAs, centromere itself also recruits proteins that repair the mismatches in DNA, such as Mlh1, Mlh3 and Pms2 (Kolas et al., 2005). All these results indicate DNA repair proteins are involved in the transactions of centromeres.

For females, their oocytes are differentiated from primordial germ cells at the middle fetus stages. Then oocytes initiate meiosis and finish meiotic homologous recombination asynchronously at the fetal stage, and arrest in the diplotene (or termed as dictyate) state and reside in the ovarian primordial follicles at the peri-birth stage (Pepling, 2006). The arrest state of oocytes may take a time as long as tens of years until they are activated for growth when the hormone environments of ovarian follicles are changed (McGee and Hsueh, 2000). The oocytes grow parallel to the development of follicles. In preantral follicles and small-antral follicles, oocytes are transcription active, and there is no Hoechst positive ring-like structure surrounded the nucleolus (NSN). When follicles develop to the late large-antral stage, the oocytes are fully grown and their transcription are globally silenced. Then there is a Hoechst positive ring-like structure surrounded the nucleolus (SN) (Tan et al., 2009). When oocytes are fully grown, they can resume meiosis (Pan and Li, 2019), extrude first polar bodies, be ovulated and fertilized by sperm (Robker et al., 2018). It has been reported that centromeric RNAs play important functions in oocyte meiosis maturation. When minSAT RNAs in oocytes were degraded, DSB marker γH2A.X will form foci at centromeres, and the interaction between kinetochore and micro-tubulin would be impaired (Wu et al., 2021). Subsequently, the maturation time and maturation rate of minSAT RNA depleted oocytes would be extended and decreased respectively (Wu et al., 2021). Although satellite RNAs play important roles during oocyte maturation, when oocyte centromeres are transcribed and which proteins are associated with the centromeric transcription are still not known.

Transcription and DNA damage in centromeres might not only affect kinetochore assembly but also induce instability of centromeres which would further result in intra-centromeric rearrangements (Wang et al., 2009; Racca et al., 2021). In this study, the transcription time of centromeres in oocytes was analyzed. DSB repair protein Mre11 and Prkdc, and mismatch repair protein Mlh1 were found localized at the transcribing centromeres. These results linked centromere transcription with DNA repair proteins, which might extend our knowledge about how oocytes assemble kinetochores and maintain centromere stability.

## Materials and methods

### Mouse oocytes collection and culture

All animal manipulations in this study were permitted by the Ethics Committee of Guangdong Second Provincial General Hospital. The oocytes used in this study were isolated from 6 to 10-week-old female mice (Zhiyuan company, ICR strain). To isolate the NSN, NSN-SN (intermediate stage between NSN and SN) and SN oocytes from antral follicles, mouse ovaries were processed firstly by blade chopping. Then the ovarian tissue fragments were dispersed by M2 medium (Sigma-Aldrich, M7167) with 2.5 μM Milrinone (MCE, HY-14252). The denuded oocytes were picked out with mouth pipette. To isolate the NSN oocytes from preantral and small-antral follicles, these follicles were picked out from the chopped ovarian fragments and treated with Trypsin-EDTA Solution (Solarbio, T1300) at 37°C for 30 min. Then the growing follicular NSN oocytes were isolated through blow-suction disturbance of the follicles by mouth pipette. To *in vitro* maturate the germinal vesicle (GV) stage oocytes, which contains NSN and SN oocytes in the late antral follicles, these oocytes were released from Milrinone by three washes in M2 medium and cultured in M2 medium in incubator (37°C, 5% CO_2_) for specific length of time.

### Immunofluorescence labeling

To label the proteins with antibodies, oocytes were firstly fixed with 4% paraformaldehyde (Sangon, E672002) at room temperature (RT) for 25 min. Then the oocyte membrane was penetrated in PBS with 0.1% Triton X-100 at RT for 25 min. Then the penetrated oocytes were treated with Quick Antigen Retrieval Solution (Beyotime, P0090) at RT for 30 min and washed with PBST (PBS with 1% Tween-20) twice, blocked by 1% BSA at RT for 1 h and incubated with primary antibodies at 4°C overnight. After washing and labelling of fluorescent molecule coupled secondary antibody, the oocytes were stained with Hoechst 33342 and mounted on slides. The antibodies used in this study were: anti-γH2A.X (Bioworlde, BS4760), anti-Mlh1 (Clone ID: 2B3-H6-C11, Zen-bio, 200956), anti-Mre11 (Sangon, D122698), anti-Prkdc (Sangon, D261628). To label the centromeres with anti-centromere protein antibody (ACA, antibodiesinc, 15–234), oocytes were not treated with Antigen Retrieval Solution. When oocytes were needed to be labelled by both ACA and other antibodies, oocytes are firstly labeled with ACA, then oocytes were refixed again. Then the oocytes were antigen-retrieved and labeled with the other antibodies as described above.

The immunofluorescence labeling results were observed by Andro dragonfly live cell work station. The nucleus or the whole oocytes were scanned by the Z-scan methods. The distance between each two layers was 1 μm. Raw image files and 3D object volumes were analyzed by Fiji software (Schindelin et al., 2012), and the single layers of the Z-scan images were displayed in this study.

### Chemical drug treatment

To inhibit the Mre11 activity, 100 μM Mirin (MCE, HY-117693) was used to treat the oocytes. GV oocytes were firstly blocked in Milrinone and treated with Mirin for 12 h and then released from Milrinone for *in vitro* maturation (IVM). The GV broken down (GVBD) rates of oocytes [N (GVBD oocytes)/N (total oocytes)] were recorded from IVM time 1 h–4 h, and the polar body extrusion (PBE) rates [N(PBE oocytes)/N (total oocytes)] were recorded from IVM 10 h–14 h. 60 μg/mL of α-amanitin (MCE, HY-19610) was used to inhibit the transcription activity of RNAPII in NSN oocytes. Oocytes were treated with 1 μM of Bleomycin (Selleck, S1214) for 1 h to induce nuclear DSBs. Medium with DMSO (0.5% v/v, MCE, HY-Y0320) was used as control for the chemical drug treatment.

### 5-EU click reaction

The click reaction of 5-EU was performed according to the instruction of BeyoClick EdU-647 kit (Beyotime, C0081S) with 5-EU (Click Chemistry Tools, 1261-10) but not the 5-ethynyl-2' -deoxyuridine. In detail, 0.5 mM 5-EU was used to treat the NSN oocytes for 3 h. Then oocytes were picked out for fixation and click reaction. If the transcription of oocytes needs to be inhibited, oocytes were treated with α-amanitin or DMSO for 3 h and then 0.5 mM 5-EU was added for another 3 h. Then oocytes were picked out for click reaction.

### Statistics

In this study, the R (https://www.r-project.org/) functions for *t*-test and Fisher exact test were used to examine the significance of differences between sample means or oocyte rates and proportions. *p* values less than 0.01 were marked by **; *p* values less than 0.05 were marked by *; and *p* values larger than 0.05 were marked by ns.

## Results

### Transcription of centromeres in mouse oocytes

**FIGURE 1:**
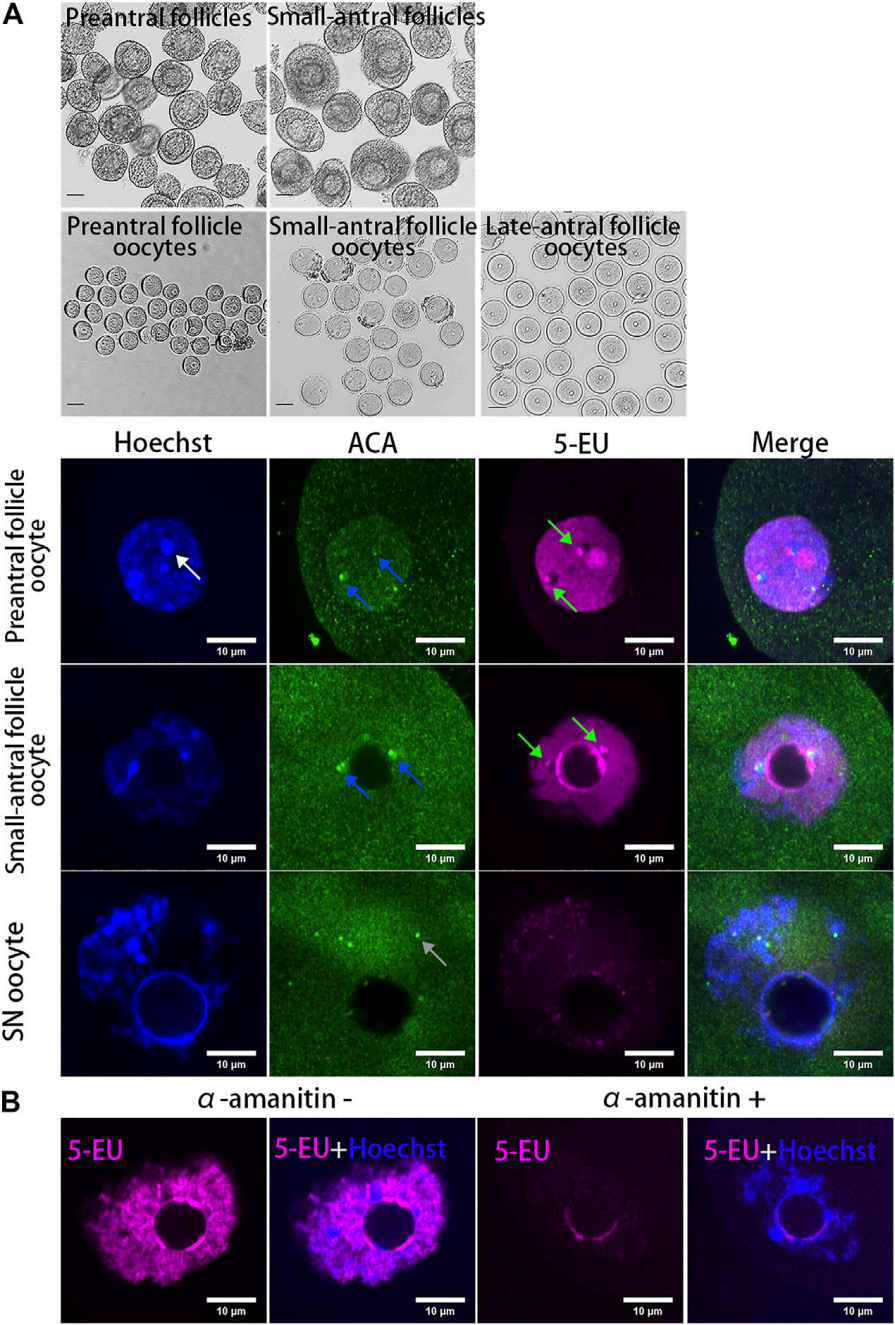
Dynamics of centromeric transcription in mouse oocytes. **(A)** Centromeres are transcribed in growing oocytes but not in fully grown oocytes. Oocytes isolated from preantral follicles and small-antral follicles are growing oocytes. SN oocytes are fully grown oocytes. New synthesized RNAs are marked by 5-EU (magenta). Minor centromeres are labeled by ACA antibodies (green). **(B)** Centromeric transcription in oocytes can be inhibited by α-amanitin. DNA are stained by Hoechst (Blue). Centromeric RNAs are indicated by green arrow. Minor satellite is indicated by blue arrow. Major satellite is indicated by white arrow. Black scale bars, 50 μm; white scale bars 10 μm.

To analyze whether the centromeres in oocytes are transcribed, mouse oocytes at different growing stages (NSN oocytes from preantral and small-antral follicles and SN oocytes from late-antral follicles) were isolated and the new synthesized RNAs were labeled with fluorescent molecules by 5-EU click reaction. As a result, we found the transcription of centromeres was highly active in growing oocytes but it was silenced in the fully grown oocytes (Figure 1A). These centromere associated RNA signals are either overlapped or distributed beside the centromeres marked by ACA antibody (Supplementary Figure S1A), which specifically recognizes the minor satellites in mouse cells (Vig et al., 1994; Guenatri et al., 2004). When oocytes were treated with the RNAPII elongating inhibitor α-amanitin, the chromatin in growing oocytes was condensed and transcription in the non-nucleolus region was inhibited, and the 5-EU labelled centromere associated RNAs were disappeared (Figure 1B).

### Mre11, prkdc and Mlh1 locate on centromeres of the growing oocytes

**FIGURE 2:**
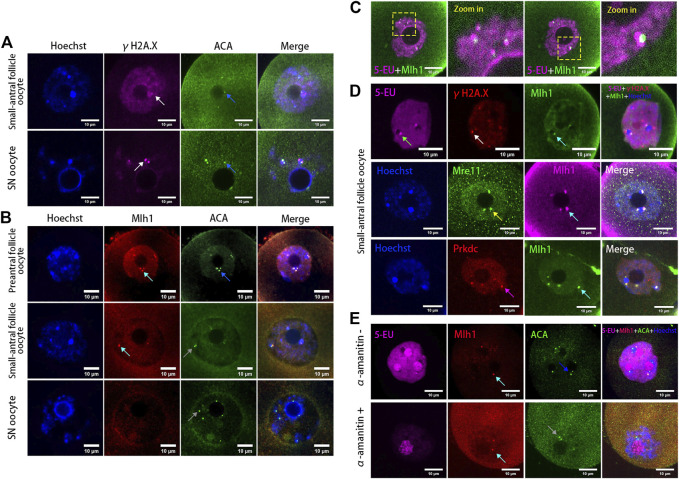
DNA repair proteins associated with centromeres in oocytes. **(A)** Minor centromeres (ACA) are not colocalized or fully overlapped with the DNA double-strand break marker γH2A.X foci. **(B)** Mlh1 (cyan arrows) colocalized with minor satellites (marked by ACA) whereas minor satellites (grey arrows) are not all colocalized with Mlh1, especially in SN oocytes (grey arrows). **(C)** Mlh1 foci in growing oocytes are associated with the centromeric RNAs. **(D)** Mlh1 foci colocalized with Mre11 and Prkdc, but not with γH2A.X foci in growing oocytes. **(E)** α-amanitin treatment induces the separation of Mlh1 foci from centromeres. Minor satellites, blue arrows; Mlh1, cyan arrows; centromeric RNAs, green arrow; Mre11 focus, yellow arrow; Prkdc focus, magenta arrow; released Mlh1 focus, red arrow; scale bars, 10 μm. DNA are stained by Hoechst (Blue).

It has been reported that human alpha-satellite RNAs can bind with key DBS repair proteins such as MRE11 and PRKDC (Zhu et al., 2018) and centromeres can bind with mismatch repair protein Mlh1 (Kolas et al., 2005). To analyze whether the centromeric transcripts in mouse oocytes are associated with DNA repair proteins, we analyzed the localization of Mre11, Prkdc, Mlh1 and the DNA DSB marker γH2A.X in the growing oocytes. As a result, we found γH2A.X foci were either not colocalized with ACA foci or not fully overlapped with ACA foci (Figure 2A), similar with our previous report (Ma et al., 2019). In contrast with γH2A.X, the Mlh1 proteins were found forming foci and localizing at the minor satellite regions marked by ACA (Figure 2B). These Mlh1 protein foci were also associated with the newly synthesized centromere associated RNAs (Figure 2C). Mre11 and Prkdc were also colocalized with ACA (Supplementary Figure S2) but not γH2A.X foci (Figure 2D). And when the transcription of oocytes was inhibited by α-amanitin, the Mlh1 foci did not disappear, however, they are unloaded from the centromeres (Figure 2E and Supplementary Figure S1B).

**FIGURE 3:**
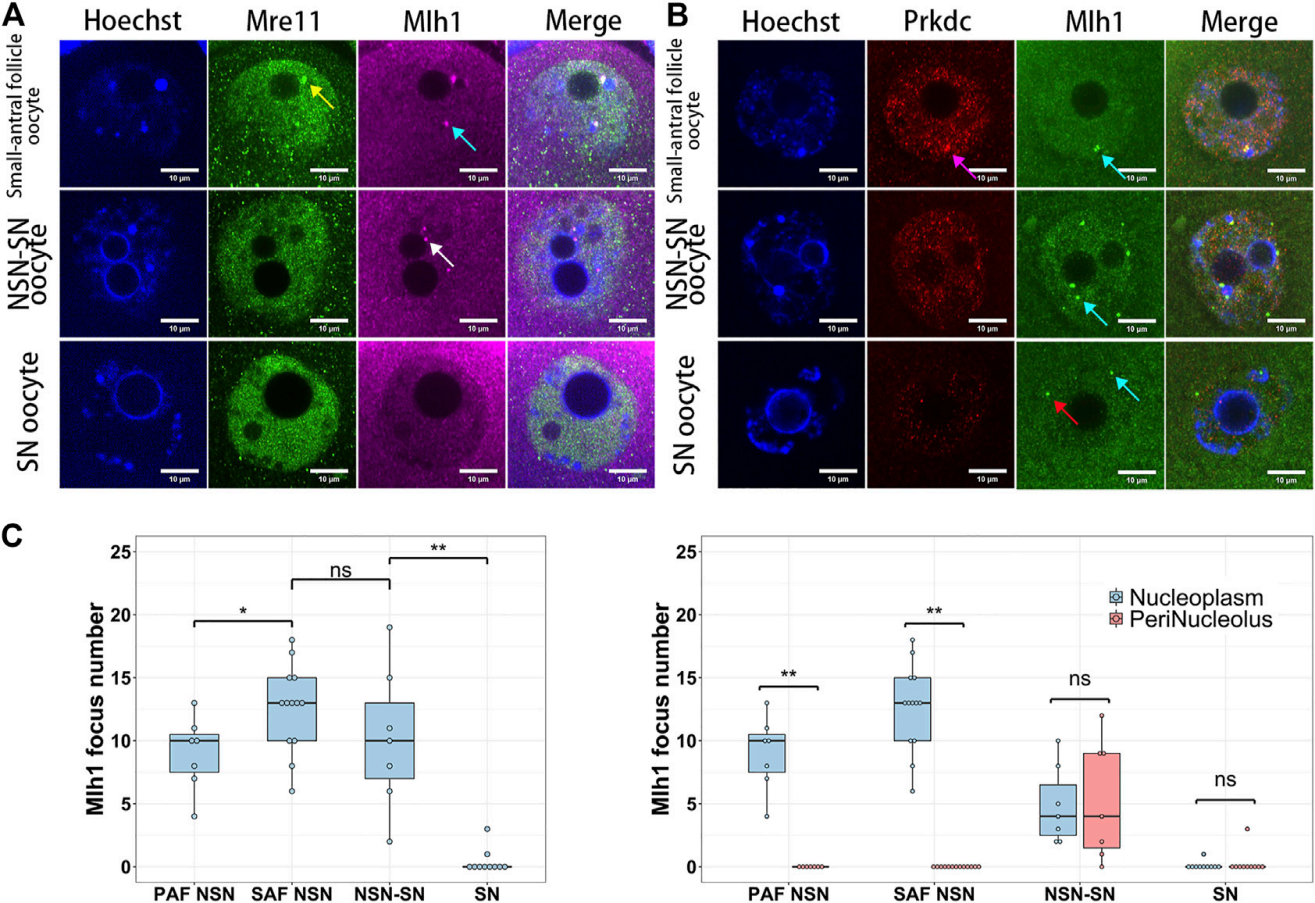
The dynamics of DNA repair proteins in oocytes. Both Mre11 and Prkdc formed focus cannot be detected in the NSN-SN or SN oocytes, but Mlh1 foci can be detected in NSN-SN oocytes and some SN oocytes **(A** and **B)**. **(C)** The statistics of Mlh1 focus number in growing and fully grown oocytes. In NSN-SN oocytes, Mlh1 foci can migrate to the peri-nucleolus regions (white arrow). Mre11 focus, yellow arrow; Prkdc focus, magenta arrow; Mlh1 foci, cyan arrows; scale bars, 10 μm; *, 0.01 < *p* < 0.05; **, *p* < 0.01; ns, non-significant. DNA are stained by Hoechst (Blue).

When mouse oocytes developed to the fully grown stage (or SN stage), the transcription was globally silenced (Tan et al., 2009) and the centromeric transcription could not be detected by 5-EU click reaction (Figure 1A). Correspondingly, foci of Mlh1, Mre11 and Prkdc were also disappeared or unloaded from chromatin. In the oocytes which were at the development stage between NSN and SN, termed NSN-SN stage, we found Mlh1 foci can still be detected but Mre11 and Prkdc focus could not be observed (Figures 3A,B), indicating that different DNA repair proteins might have different affinities with centromeres. During the growth of NSN oocytes from preantral follicles to small-antral follicles, the foci numbers of Mlh1 were increased (*p* = 0.015 by *t*-test). But during the oocytes developed from NSN-SN stage to SN stage, we found Mlh1 foci numbers were significantly decreased to zero (8/10 oocytes, *p* = 3.518e-05 by *t*-test) (Figure 3C). And only in the NSN-SN stage, Mlh1 foci can be found at the perinucleolus regions (indicated by white arrow in Figure 3A and the numbers were showed in Figure 3C).

### Meiosis resumption of NSN oocytes releases Mlh1/Mre11/prkdc foci to ooplasm

**FIGURE 4:**
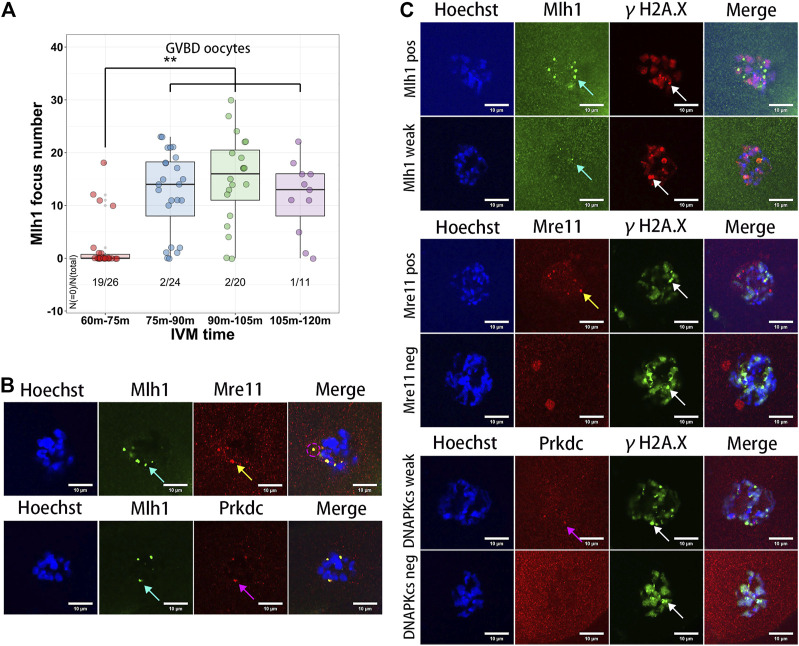
Meiosis resumption of growing oocytes released DNA repair protein foci to ooplasm. **(A)** Mlh1 focus number in the oocytes which have GVBD at different IVM time. GVBD, germinal vesicle breakdown; IVM, *in vitro* maturation. **(B)** The foci formed by DNA repair proteins are released to the ooplasm in the meiosis resumed oocytes (indicated by magenta dashed circle). **(C)** The released DNA repair protein foci are not colocalized with γH2A.X foci in the GVBD oocytes. Mre11 foci, yellow arrows; Prkdc foci, magenta arrows; Mlh1 foci, cyan arrows; γH2A.X foci, white arrows; scale bars, 10 μm. DNA are stained by Hoechst (Blue).

Among the technologies of assisted reproduction in animals and human, IVM of oocytes is frequently used for getting matured oocytes through *in vitro* culturing of the germinal vesicle stage oocytes (Hatirnaz et al., 2018; De Vos et al., 2021). During the IVM of oocytes, both growing and fully grown oocytes can undergo GVBD, develop to MII stage, and be fertilized. But the maturation rates and embryo developmental competence of the growing oocytes are lower than that of the fully grown oocytes (Bellone et al., 2009). In addition, the GVBD time of the growing oocytes is longer than that of the fully grown oocytes (Belli et al., 2014). To analyze the dynamics of the minor satellite-localized DNA repair protein foci during IVM, we obtained the GVBD oocytes at different IVM times (60–75 min, 75–90 min, 90–105 min and 105–120 min). As a result, the Mlh1 foci were found in the ooplasms of GVBD oocytes. And the Mlh1 foci were mainly found in the GVBD delayed oocytes (GVBD at 75–120 min, 50/55), which correspond to the growing NSN oocytes, but rarely be found in the earlier GVBD oocytes, which correspond to the fully grown oocytes (GVBD at 60–75 min, 7/26, *p* = 1.027e-08 by Fisher’s exact test) (Figure 4A). In the Mlh1 positive GVBD oocytes, these Mlh1 foci were found be released from the condensed chromosomes (Figure 4B). In addition, Mre11 and Prkdc foci, like Mlh1 foci, were also found in the ooplsm of oocytes which spent longer time for GVBD and all these protein foci were not colocalized with the γH2A.X foci (Figure 4C).

### Mre11 inhibition blocks the GVBD of growing oocytes

**FIGURE 5:**
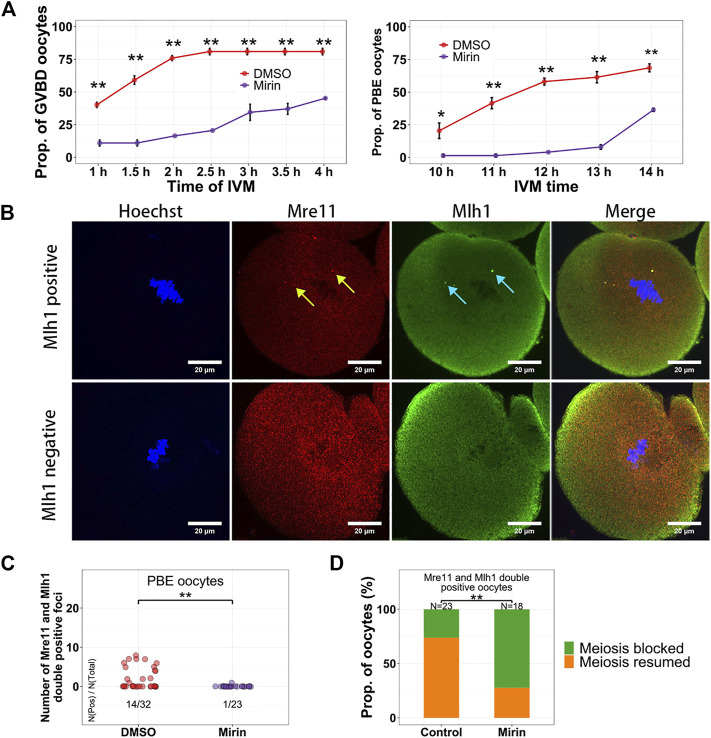
Mirin suppresses the meiosis resumption of growing oocytes. **(A)** Oocytes were treated with Mirin for 12 h in Milrinone, and then released from Milrinone for *in vitro* maturation. GVBD and PBE of oocytes are suppressed by Mirin. **(B)** Mlh1 and Mre11 foci can be detected in the MII (metaphase of the second meiosis) oocytes. **(C)** Compared with control (DMSO) group, the Mre11 and Mlh1 focus positive PBE oocytes are decreased in the Mirin treated group. **(D)** For the Mre11 and Mlh1 positive oocytes, more Mirin treated oocytes are meiosis-blocked. Mre11 focus, yellow arrow; Mlh1, cyan arrow; *, 0.01 < *p* < 0.05; **, *p* < 0.01; scale bars, 10 μm. DNA are stained by Hoechst (Blue).

In these centromeric localized proteins, Mre11 is a nuclease acting on various DNA substrates like DSB ends, overhangs, flaps, branches, and non-B-DNA structures like hairpins and ring structures (D'Amours and Jackson, 2002). In addition, the Mre11/Rad50/Nbs1 complex is the sensor of DSBs and can recruit DNA damage transductors like ATM to regulate cell cycle (Lavin, 2007). In activated B lymphocyte, it has been reported that non–B-DNA structures like cruciforms and hairpins can form at the minor satellites (Kasinathan and Henikoff, 2018), which might be potential substrates of Mre11. It has been reported that the Mre11 inhibitor Mirin can suppress DSB repair and decrease maturation rate of oocytes (Mayer et al., 2016). However, whether Mirin affects Mre11 localization and which oocytes are affected by Mirin during their maturation are still unknown. When oocytes were treated with Mirin and blocked in Milrinone for 12 h, we found the localization of both Mre11 and Mlh1 had not been changed (Supplementary Figure S3A). When oocytes were treated with Bleomycin to induce DSBs and released from Bleomycin for 6 h, the volume of DSB marker γH2A.X were significantly larger in Mirin treated oocytes than that in DMSO treated control oocytes (Supplementary Figure S3B). For the oocytes which were only treated with Mirin, as reported previously (Mayer et al., 2016), after releasing from Milrinone, both GVBD time and PBE time of these oocytes were delayed (Figure 5A). In the control oocytes which had developed to MII stage, Mre11 foci and Mlh1 foci could still be detected. However, in the PBE oocytes treated with Mirin, the proportion of oocytes with Mlh1 and Mre11 overlapped foci were much less (1/23 for Mirin treated oocytes vs 14/32 for control oocytes, *p* = 0.001572 by Fisher’s exact test) (Figures 5B,C). For the oocytes with Mlh1 and Mre11 double positive foci, 73.9% of control oocytes (17/23) had GVBD whereas only 27.8% of Mirin treated oocytes (5/18) had GVBD (*p* = 0.00487 by Fisher’s exact test) (Figure 5D), indicating that Mirin suppressed the GVBD of NSN oocytes with Mre11 foci.

## Discussion

Centromeric transcription is not only essential for kinetochore assembly but also associated with genome stability. In this study, we found the transcription of centromeres in mouse growing oocytes and its relationships with DNA repair proteins. These results indicate that there might be links between centromere transcription and DNA repair. Regional centromeres are generally composed of kilobases to megabases of repeats like satellites and dispersed elements. These repeats contribute to the complex 3-D organization of centromeres (Navarro and Cheeseman, 2021). Under the supercoiling stress of transcription (Ma and Wang, 2016), centromeres may produce different types of DNA structures (Kasinathan and Henikoff, 2018) as well as mismatches between repeats. In the centromere associated proteins, both Mre11 and Prkdc can take functions on specific DNA structures (D'Amours and Jackson, 2002; Fan et al., 2022) and Mlh1 is a key player for mismatch repair. The loading of these DNA repair proteins to centromeres might be beneficial for the maintenance of centromere stability.

When meiosis was resumed in the NSN oocytes, the Mlh1/Mre11/Prkdc foci on centromeres were released into the cytoplasm. This result indicates that these DNA repair foci may not bind with centromere DNA, and can be released when the oocyte is going to pro-metaphase. Evidences had proven that Mre11 can interact with RNAPII directly or mediated by PIH1D1 (Michelini et al., 2017; von Morgen et al., 2017; Sharma et al., 2021). And Prkdc can also interact and phosphorylate the RNAPII (Dvir et al., 1992; Maldonado et al., 1996). There is no evidence about the interaction between Mlh1 and RNAPII, but Mlh1 can bind with Mre11 (Her et al., 2002; Mirzoeva et al., 2006). The above evidences and findings indicated that the centromere loading of Mre11/Mlh1/Prkdc proteins might be mediated by the transcription machinery. However, whether the centromere localization of these DNA repair proteins directly or indirectly depends on centromeric transcription is still an open question.

In addition, in our results we found these Mre11 and Mlh1 foci released from chromatin can be preserved at least at the MII oocyte stage. This evidence indicates the proteins on these foci might be insoluble and lack DNA repair functions. In addition, these proteins could only be observed by immunofluorescence labeling when the oocytes were treated with antigen retrieval reagents which contain the protein denaturant sodium dodecyl sulfate, indicating that the 3D structures of these centromere associated proteins might be abnormal.

It has been reported that Mre11 can promote the DSB repair in oocytes (Mayer et al., 2016). In the last result of this study, we found the Mre11 inhibitor Mirin could block the meiosis resumption of NSN oocytes but it did not impair the centromere localization of Mre11. Whether Mirin could block the biological functions of these centromeric Mre11 is not known. Nevertheless, the recruitment of DNA repair proteins at centromeres would sequestrate these DNA repair proteins (Zhu et al., 2018) and may impact the DNA damage response in oocytes.

Last but not least, in this study, the sequences of the newly synthesized centromere associated RNAs in the growing oocytes had not been identified. There is a possibility that these RNAs are not transcribed from centromeres, but from chromosome arms and migrate to the centromere region.

## Data Availability

The original contributions presented in the study are included in the article/Supplementary Material, further inquiries can be directed to the corresponding authors.

## References

[B1] Belli M. Vigone G. Merico V. Redi C. A. Garagna S. Zuccotti M. (2014). Time-lapse dynamics of the mouse oocyte chromatin organisation during meiotic resumption. Biomed. Res. Int. 2014, 207357. 10.1155/2014/207357 24864231 PMC4016838

[B2] Bellone M. Zuccotti M. Redi C. A. Garagna S. (2009). The position of the germinal vesicle and the chromatin organization together provide a marker of the developmental competence of mouse antral oocytes. Reproduction 138 (4), 639–643. 10.1530/REP-09-0230 19633134

[B3] Bergmann J. H. Rodriguez M. G. Martins N. M. Kimura H. Kelly D. A. Masumoto H. (2011). Epigenetic engineering shows H3K4me2 is required for HJURP targeting and CENP-A assembly on a synthetic human kinetochore. EMBO J. 30 (2), 328–340. 10.1038/emboj.2010.329 21157429 PMC3025471

[B4] Chen S. Lees-Miller J. P. He Y. Lees-Miller S. P. (2021). Structural insights into the role of DNA-PK as a master regulator in NHEJ. Genome Instab. Dis. 2 (4), 195–210. 10.1007/s42764-021-00047-w 34723130 PMC8549938

[B5] Chueh A. C. Northrop E. L. Brettingham-Moore K. H. Choo K. H. Wong L. H. (2009). LINE retrotransposon RNA is an essential structural and functional epigenetic component of a core neocentromeric chromatin. PLoS Genet. 5 (1), e1000354. 10.1371/journal.pgen.1000354 19180186 PMC2625447

[B6] D'Amours D. Jackson S. P. (2002). The Mre11 complex: At the crossroads of dna repair and checkpoint signalling. Nat. Rev. Mol. Cell. Biol. 3 (5), 317–327. 10.1038/nrm805 11988766

[B7] De Vos M. Grynberg M. Ho T. M. Yuan Y. Albertini D. F. Gilchrist R. B. (2021). Perspectives on the development and future of oocyte IVM in clinical practice. J. Assist. Reprod. Genet. 38 (6), 1265–1280. 10.1007/s10815-021-02263-5 34218388 PMC8266966

[B8] Dvir A. Peterson S. R. Knuth M. W. Lu H. Dynan W. S. (1992). Ku autoantigen is the regulatory component of a template-associated protein kinase that phosphorylates RNA polymerase II. Proc. Natl. Acad. Sci. U. S. A. 89 (24), 11920–11924. 10.1073/pnas.89.24.11920 1465419 PMC50669

[B9] Fan Y. Liang Y. Liu Y. Fan H. (2022). PRKDC promotes Hepatitis B virus transcription through enhancing the binding of RNA Pol II to cccDNA. Cell. Death Dis. 13 (4), 404. 10.1038/s41419-022-04852-3 35468873 PMC9038722

[B10] Guenatri M. Bailly D. Maison C. Almouzni G. (2004). Mouse centric and pericentric satellite repeats form distinct functional heterochromatin. J. Cell. Biol. 166 (4), 493–505. 10.1083/jcb.200403109 15302854 PMC2172221

[B11] Hatirnaz S. Ata B. Hatirnaz E. S. Dahan M. H. Tannus S. Tan J. (2018). Oocyte *in vitro* maturation: A sytematic review. Turk J. Obstet. Gynecol. 15 (2), 112–125. 10.4274/tjod.23911 29971189 PMC6022428

[B12] Her C. Vo A. T. Wu X. (2002). Evidence for a direct association of hMRE11 with the human mismatch repair protein hMLH1. DNA Repair (Amst) 1 (9), 719–729. 10.1016/s1568-7864(02)00079-4 12509276

[B13] Kasinathan S. Henikoff S. (2018). Non-B-Form DNA is enriched at centromeres. Mol. Biol. Evol. 35 (4), 949–962. 10.1093/molbev/msy010 29365169 PMC5889037

[B14] Kolas N. K. Svetlanov A. Lenzi M. L. Macaluso F. P. Lipkin S. M. Liskay R. M. (2005). Localization of MMR proteins on meiotic chromosomes in mice indicates distinct functions during prophase I. J. Cell. Biol. 171 (3), 447–458. 10.1083/jcb.200506170 16260499 PMC2171243

[B15] Lavin M. F. (2007). ATM and the Mre11 complex combine to recognize and signal DNA double-strand breaks. Oncogene 26 (56), 7749–7758. 10.1038/sj.onc.1210880 18066087

[B16] Ma J. Wang M. D. (2016). DNA supercoiling during transcription. Biophys. Rev. 8 (1), 75–87. 10.1007/s12551-016-0215-9 PMC5338639 28275417

[B17] Ma J. Y. Feng X. Tian X. Y. Chen L. N. Fan X. Y. Guo L. (2019). The repair of endo/exogenous DNA double-strand breaks and its effects on meiotic chromosome segregation in oocytes. Hum. Mol. Genet. 28 (20), 3422–3430. 10.1093/hmg/ddz156 31384951

[B18] Maldonado E. Shiekhattar R. Sheldon M. Cho H. Drapkin R. Rickert P. (1996). A human RNA polymerase II complex associated with SRB and DNA-repair proteins. Nature 381 (6577), 86–89. 10.1038/381086a0 8609996

[B19] Mayer A. Baran V. Sakakibara Y. Brzakova A. Ferencova I. Motlik J. (2016). DNA damage response during mouse oocyte maturation. Cell. Cycle 15 (4), 546–558. 10.1080/15384101.2015.1128592 26745237 PMC5056612

[B20] McGee E. A. Hsueh A. J. (2000). Initial and cyclic recruitment of ovarian follicles. Endocr. Rev. 21 (2), 200–214. 10.1210/edrv.21.2.0394 10782364

[B21] Michelini F. Pitchiaya S. Vitelli V. Sharma S. Gioia U. Pessina F. (2017). Damage-induced lncRNAs control the DNA damage response through interaction with DDRNAs at individual double-strand breaks. Nat. Cell. Biol. 19 (12), 1400–1411. 10.1038/ncb3643 29180822 PMC5714282

[B22] Mirzoeva O. K. Kawaguchi T. Pieper R. O. (2006). The Mre11/Rad50/Nbs1 complex interacts with the mismatch repair system and contributes to temozolomide-induced G2 arrest and cytotoxicity. Mol. Cancer Ther. 5 (11), 2757–2766. 10.1158/1535-7163.MCT-06-0183 17121922

[B23] Muller H. Gil J. Jr. Drinnenberg I. A. (2019). The impact of centromeres on spatial genome architecture. Trends Genet. 35 (8), 565–578. 10.1016/j.tig.2019.05.003 31200946

[B24] Navarro A. P. Cheeseman I. M. (2021). Kinetochore assembly throughout the cell cycle. Semin. Cell. Dev. Biol. 117, 62–74. 10.1016/j.semcdb.2021.03.008 33753005 PMC8384650

[B25] Pan B. Li J. (2019). The art of oocyte meiotic arrest regulation. Reprod. Biol. Endocrinol. 17 (1), 8. 10.1186/s12958-018-0445-8 30611263 PMC6320606

[B26] Pepling M. E. (2006). From primordial germ cell to primordial follicle: Mammalian female germ cell development. Genesis 44 (12), 622–632. 10.1002/dvg.20258 17146778

[B27] Plohl M. Mestrovic N. Mravinac B. (2014). Centromere identity from the DNA point of view. Chromosoma 123 (4), 313–325. 10.1007/s00412-014-0462-0 24763964 PMC4107277

[B28] Racca C. Britton S. Hedouin S. Francastel C. Calsou P. Larminat F. (2021). BRCA1 prevents R-loop-associated centromeric instability. Cell. Death Dis. 12 (10), 896. 10.1038/s41419-021-04189-3 34599155 PMC8486751

[B29] Robker R. L. Hennebold J. D. Russell D. L. (2018). Coordination of ovulation and oocyte maturation: A good egg at the right time. Endocrinology 159 (9), 3209–3218. 10.1210/en.2018-00485 30010832 PMC6456964

[B30] Schindelin J. Arganda-Carreras I. Frise E. Kaynig V. Longair M. Pietzsch T. (2012). Fiji: An open-source platform for biological-image analysis. Nat. Methods 9 (7), 676–682. 10.1038/nmeth.2019 22743772 PMC3855844

[B31] Sharma S. Anand R. Zhang X. Francia S. Michelini F. Galbiati A. (2021). MRE11-RAD50-NBS1 complex is sufficient to promote transcription by RNA polymerase II at double-strand breaks by melting DNA ends. Cell. Rep. 34 (1), 108565. 10.1016/j.celrep.2020.108565 33406426 PMC7788559

[B32] Talbert P. B. Henikoff S. (2018). Transcribing centromeres: Noncoding RNAs and kinetochore assembly. Trends Genet. 34 (8), 587–599. 10.1016/j.tig.2018.05.001 29871772

[B33] Tan J. H. Wang H. L. Sun X. S. Liu Y. Sui H. S. Zhang J. (2009). Chromatin configurations in the germinal vesicle of mammalian oocytes. Mol. Hum. Reprod. 15 (1), 1–9. 10.1093/molehr/gan069 19019837

[B34] Ting D. T. Lipson D. Paul S. Brannigan B. W. Akhavanfard S. Coffman E. J. (2011). Aberrant overexpression of satellite repeats in pancreatic and other epithelial cancers. Science 331 (6017), 593–596. 10.1126/science.1200801 21233348 PMC3701432

[B35] Vig B. K. Latour D. Frankovich J. (1994). Dissociation of minor satellite from the centromere in mouse. J. Cell. Sci. 107 (11), 3091–3095. 10.1242/jcs.107.11.3091 7535306

[B36] von Morgen P. Burdova K. Flower T. G. O'Reilly N. J. Boulton S. J. Smerdon S. J. (2017). MRE11 stability is regulated by CK2-dependent interaction with R2TP complex. Oncogene 36 (34), 4943–4950. 10.1038/onc.2017.99 28436950 PMC5531254

[B37] Wang J. C. Hajianpour A. Habibian R. (2009). Centromeric alpha-satellite DNA break in reciprocal translocations. Cytogenet Genome Res. 125 (4), 329–333. 10.1159/000235939 19864896

[B38] Wu T. Lane S. I. R. Morgan S. L. Tang F. Jones K. T. (2021). Loss of centromeric RNA activates the spindle assembly checkpoint in mammalian female meiosis I. J. Cell. Biol. 220 (10), e202011153. 10.1083/jcb.202011153 34379093 PMC8360762

[B39] Zhao F. Kim W. Kloeber J. A. Lou Z. (2020). DNA end resection and its role in DNA replication and DSB repair choice in mammalian cells. Exp. Mol. Med. 52 (10), 1705–1714. 10.1038/s12276-020-00519-1 33122806 PMC8080561

[B40] Zhu Q. Pao G. M. Huynh A. M. Suh H. Tonnu N. Nederlof P. M. (2011). BRCA1 tumour suppression occurs via heterochromatin-mediated silencing. Nature 477 (7363), 179–184. 10.1038/nature10371 21901007 PMC3240576

[B41] Zhu Q. Hoong N. Aslanian A. Hara T. Benner C. Heinz S. (2018). Heterochromatin-encoded satellite RNAs induce breast cancer. Mol. Cell. 70 (5), 842–853.e7. 10.1016/j.molcel.2018.04.023 29861157 PMC6545586

