# Supplementary material for: Dynamic of centromere associated RNAs and the centromere loading of DNA repair proteins in growing oocytes
Source: Front Genet. 2023 Mar 24;14:1131698. doi: 10.3389/fgene.2023.1131698 (PMC10080056; doi:10.3389/fgene.2023.1131698)
Supplement: Supplementary file 1 [file Presentation1.pdf]

## Supplementary Figure legends

**Figure S1. Z-scan images of the newly synthesized RNAs, centromeres and Mlh1 foci.** (**A**) Z-scan images of the newly synthesized RNAs (5-EU) and centromeres (marked by anti-centromere antibody ACA). (**B**) Z-scan images of DMSO or α-amanitin treated oocytes.

**Figure S2. Mre11 and Prkdc colocalize with centromeres in growing oocytes.** (**A**) Mre11 forms foci and colocalizes with centromeres (marked by ACA) in growing oocytes (NSN oocytes) but not in fully grown oocytes (SN oocytes). (**B**) Prkdc shows a similar expression and localization pattern with Mre11 in oocytes.

**Figure S3. Mirin doesn't affect the localization of Mre11 but affects DSB repair in oocytes.** (**A**) After Mirin (100 μM) treatment for 12 hours, both Mre11 and Mlh1 foci are still close to the centromeres (marked by the Hoechst positive plaques). (**B**) Mirin suppressed the DSB repair procession in oocytes. The Milrinone blocked oocytes were treated with 100 μM Mirin (DMSO was used as control) for 12 hours, and then treated by 1 μM Bleomycin for 1 hour and released from Bleomycin for 6 hours. γH2A.X volumes were calculated by FIJI software. **, $p < 0.01$; scale bars, 10 μm.
