# Supplementary figures and images for: Dynamic of centromere associated RNAs and the centromere loading of DNA repair proteins in growing oocytes
Source: Front Genet. 2023 Mar 24;14:1131698. doi: 10.3389/fgene.2023.1131698 (PMC10080056; doi:10.3389/fgene.2023.1131698)

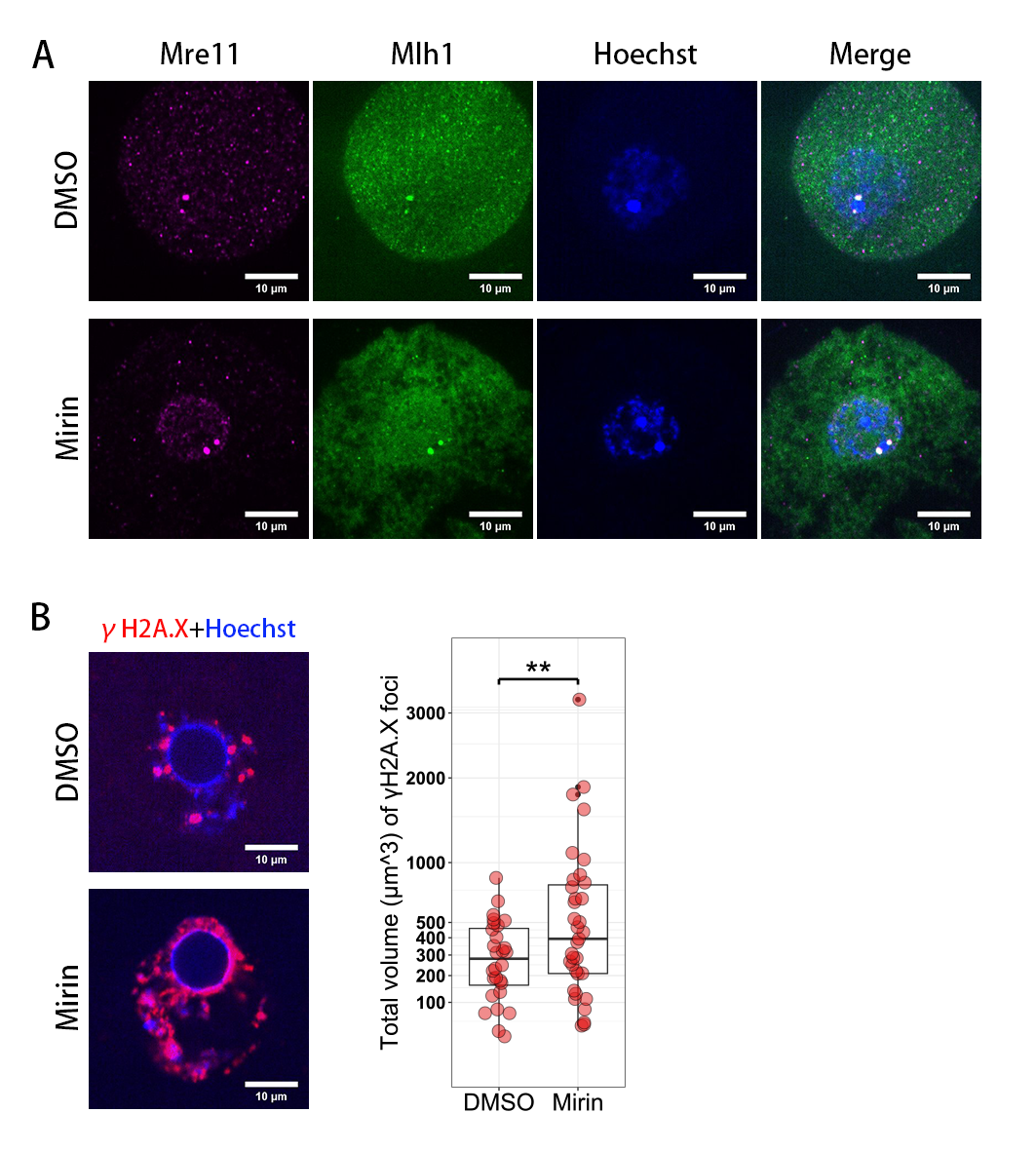

Supplement: Supplementary file 2 [file Image3.TIF]

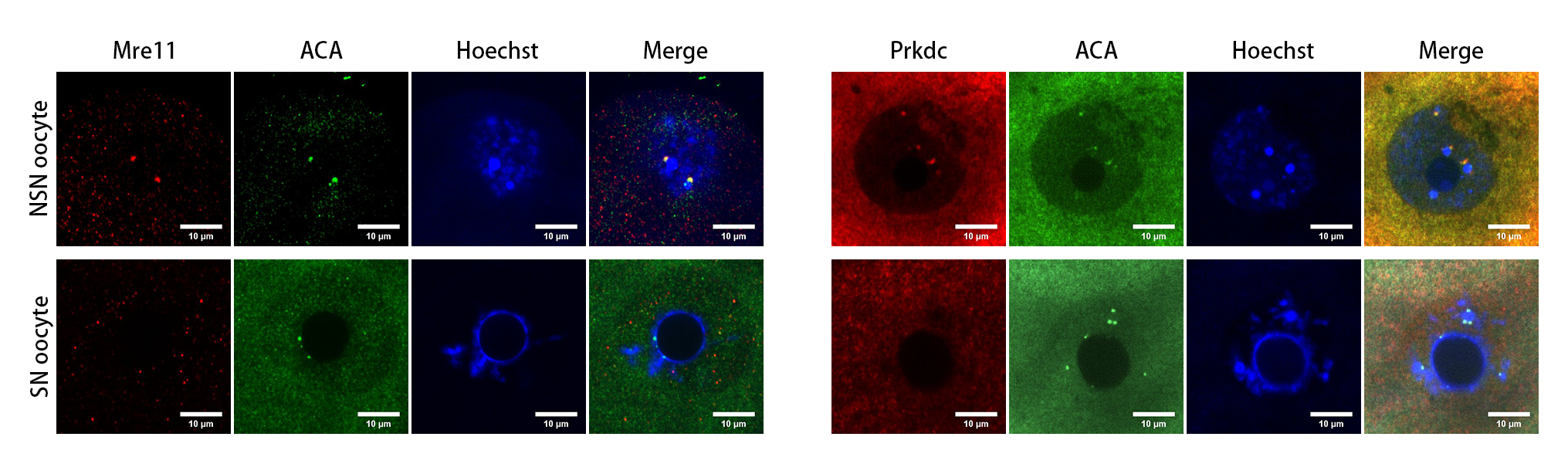

Supplement: Supplementary file 3 [file Image2.TIF]

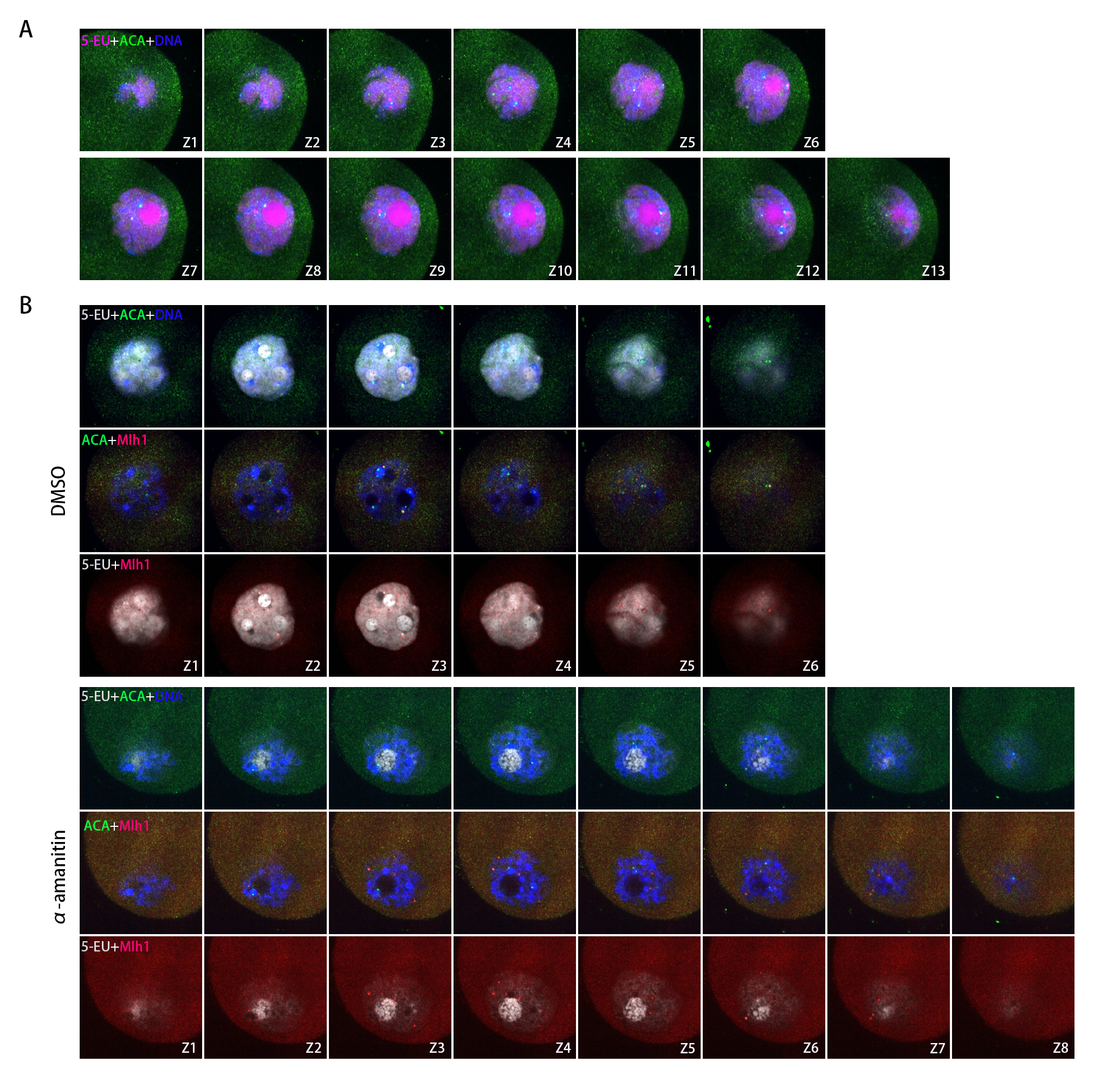

Supplement: Supplementary file 4 [file Image1.TIF]
